# Risk Factors and Management for Epistaxis in a Hospitalized Adult Sample

**DOI:** 10.51894/001c.37760

**Published:** 2022-09-06

**Authors:** Andrew Ross, Steven Engebretsen, Rebecca Mahoney, Samba Bathula

**Affiliations:** 1 Otolaryngology-Head and Neck Surgery Detroit Medical Center

**Keywords:** Epistaxis, inpatient population, treatment of epistaxis, prevention of epistaxis

## Abstract

**INTRODUCTION:**

Epistaxis is a common otolaryngologic problem that affects most of the general population. Common risk factors for epistaxis include nasal irritants, nasal/facial oxygen use, certain systemic conditions (e.g., hypertension and coagulopathies) and medication use (e.g., anticoagulants and intranasal medications). This study examined risk factors for and management of epistaxis in patients admitted for other medical conditions who developed an episode of epistaxis during their hospital admission.

**METHODS:**

Patients were included in the study if they were older than 18, admitted for medical illnesses other than epistaxis and developed an episode of epistaxis during their admission during calendar year 2020 at the authors’ institution’s hospitals. Electronic health record data regarding sociodemographic characteristics, common risk factors (e.g. oxygen use, anticoagulant use, history of hypertension) and treatment for epistaxis (e.g. holding anticoagulation therapy, administration of oxymetazoline, nasal cautery, nasal packing) were extracted from each chart. Patients were split into otolaryngologic treatment versus no treatment groups and risk factors were compared between sample subgroups.

**RESULTS:**

A total of 143 sample patients were included, with most common reason for admission being cardiovascular related, 48 (33.6%). Most patients, 104 (72.7%), did not have a previous diagnosis of epistaxis, were positive for anticoagulant use, 106 (74.1%) and were positive for hypertension, 95 (66.4%). Oxygen use showed a significantly decreased risk for intervention (OR 0.45, 95% CI: 0.23-0.894; p = 0.028). Most patients required changes in medical management (e.g., holding anticoagulation or starting nasal saline sprays/emollients).

**CONCLUSION:**

These results demonstrate the common risk factors for epistaxis in patients admitted for other clinical diseases. Identifying at-risk patients for epistaxis at hospital admission can help to initiate measures to prevent epistaxis episodes. Future studies are needed to study epistaxis risk factors and identify effective preventative measures for epistaxis among hospital populations.

## INTRODUCTION

Epistaxis (i.e., nose bleed) is a common otolaryngologic problem that affects about 60% of the general population.^1^ Only about 6% of patients with epistaxis episodes will seek medical attention.^2^ Although approximately 0.5% of epistaxis cases are severe enough to present to the emergency department, only 0.2% have been estimated to require hospitalization.^2,3^ There appears to be a bi-modal frequency related to age with epistaxis being common in patients younger than 10 and between the ages of 70-79.^2,3^

Although most epistaxis cases begin spontaneously, some can also be due to trauma.^2–4^ Typical risk factors for epistaxis include facial injury, physical or chemical mucosal irritation, allergic rhinitis, infectious rhinitis, nasal tumors, temperature, and humidity.^2,3,5^ Other systemic conditions (e.g., hypertension, coagulation disorders, diabetes, heart failure, anemia, liver disorders) factors can also play a role in the development of epistaxis as well as certain medication use (i.e., anticoagulants, intranasal medications).^5,6^

Epistaxis most commonly occurs in the anterior portion of the nose at Kiesselbach’s plexus which is a confluence area of five different blood vessels. Bleeding can also occur posteriorly from Woodruff’s plexus which is composed of branches of the sphenopalatine and posterior ethmoidal arteries which are both main vessels that provide the blood supply to the back of the nose or other portions of the nasal cavity not visible on anterior nasal examination.^6^

Initial management of an epistaxis patient includes estimating blood loss, assessing volume status, obtaining intravenous access, and checking for coagulopathies. First-aid measures include instructing the patient to apply constant, firm pressure to the lower cartilaginous portion of the nose for 20 minutes. If topical decongestants, such as oxymetazoline HCl, are available, they should be sprayed into the nose.^7^ The nasal cavity should be cleared of any clots, inspected for the source of bleeding, and silver nitrate cautery may be used if the bleeding source is identified.^7^ If the bleeding source cannot be identified or controlled, nasal endoscopy or packing is required for further management.^7–9^

Prevention strategies for epistaxis include humidification of supplemental oxygen, nasal emollients (e.g., petroleum jelly, antibiotic ointment, saline gel) and nasal saline sprays.^2,9^ Previous studies have examined patients who present to office-based clinics, emergency departments or are admitted to hospitals solely for epistaxis management.^3–5,10^ Only one 2021 study has specifically investigated epistaxis risk factors in a hospitalized sample.^11^

### Study Purpose

The purpose of this retrospective cross-sectional study was to examine the significance of known risk factors for epistaxis in a sample of hospitalized patients admitted for other clinical diseases in addition to epistaxis treatment and prevention patterns. The primary outcome of this study was to identify risk factors for epistaxis of hospitalized patients who were admitted for other medical conditions and determine if any significant risk factors increased the need for otolaryngologic intervention.

## METHODS

Using electronic health record (EHR) data, the study team sampled hospitalized patients admitted during calendar year 2020 who were diagnosed with epistaxis based on their ICD-10 codes. Before data collection, the study was approved by the Detroit Medical Center and Wayne State University institutional review boards. Inclusion criteria were patients >18-years-old currently admitted to one of the authors’ four system hospitals with a diagnosis of epistaxis during their hospital stay for a reason unrelated to their admitting diagnosis.

Documentation of the epistaxis episode(s) and treatment decisions were required to be included in the study sample. Patients were excluded if they were admitted to the hospital for epistaxis, had recent nasal trauma, had no active bleeding during their hospital stay or underwent nasal surgery in the past 30 days.

EHR data concerning patient sociodemographic characteristics, admission diagnoses, identifiable epistaxis risk factors, and treatments were extracted from patients’ medical records. Specific evaluated risk factors for epistaxis included nasal/facial oxygen use, anticoagulation, and medical comorbidities (e.g., coagulopathies, hypertension, prior epistaxis, diabetes, hepatic, or renal disease). Tobacco and ethanol use were also recorded. Based on provider documentation, sample patients were separated into two sample subgroups.

The first group was termed the ‘intervention’ group and included otolaryngology care (e.g., nasal cautery, nasal packing, or surgical control of hemorrhage). The second group was termed ‘no intervention’ and included patients with self-limiting epistaxis that stopped spontaneously or with manual pressure.

Second author SE performed the analysis for this study. SPSS (IBM, Armonk, NY) was used for data analysis. Kolmogorov-Smirnov tests for normality were performed followed by Independent-Samples Mann-Whitney U Test for continuous variables (e.g., age). Chi square and Fisher’s exact test were used for categorical variables which included the primary outcome (i.e., epistaxis risk factors). The de-identified working data from this study are available from the corresponding author upon reasonable request.

## RESULTS

Two-hundred eighty-one patients with a diagnosis of epistaxis were first identified. 138 (49.1%) patients were excluded for the following reasons: five (3.6%) patients with recent nasal surgery, 42 (30.4%) with nasal trauma, 63 (45.7%) with no nasal bleeding during admission, and 28 (20.3%) who presented to the hospital with documented nasal bleeding. Sample patient characteristics are described in Table 1. Average age was 58 (SD = 14.9), range: 22-88. The sample was evenly split by gender with 72 (50.3%) male and 71 (49.7%) female. The most common reported Racial Affiliation characteristic was African American, 101 (70.6%). The most common reason for admission was cardiovascular related, 48 (33.6%).

**Table 1.** Characteristics of study population

	**N (%)**
**Gender**	
**Male**	72 (50.3)
**Female**	71 (49.7)
**Racial Affiliation**	
African American	101 (70.6)
Unknown	22 (15.4)
Caucasian	14 (9.8)
Cuban	4 (2.8)
Middle Eastern	2 (1.4)
**Admission Diagnosis (Top three)**	
Cardiovascular	48 (33.6)
Infectious	12 (8.4)
Hematology	10 (7.0)

Risk factors for epistaxis are recorded in Table 2. Most, 104 (72.7%), sample patients did not receive a previous epistaxis diagnosis, 106 (74.1%) patients were using anticoagulant medications and 95 (66.4%) patients had a hypertension diagnoses.

**Table 2.** Study sample risk factors

**Risk Factor**	**N (%)**
**AC/AP Use**	106 (74.1)
**Hypertension Diagnosis**	95 (66.4)
**Tobacco**	74 (51.7)
**Nasal Decongestant Use**	69 (48.3)
**Oxygen Use**	71 (49.7)
**Alcohol**	63 (44.1)
**Renal Disease**	55 (38.4)
**Previous Epistaxis **	39 (27.3)
**Liver Disease **	19 (13.3)

AC/AP(Anticoagulation/Antiplatelet)

### Intervention vs. Non-Intervention Subgroup Comparisons

There were 60 (42%) patients in the intervention group and 83 (58%) patients in the no intervention group. Age was not normally distributed based on normality testing and was not significantly different between sample subgroups (Intervention; Mean 59.83 (SD=13.6), No Intervention; Mean 58.1 (SD=15.9), p = 0.827). Neither was Gender (Intervention; Female= 24 (16.8%), Male= 36 (25.2%), No Intervention; Female= 47 (32.9%), Male= 36 (25.2%), p = 0.063) or Racial Affiliation (Intervention; African American= 42 (29.4%), Caucasian=4 (2.8%), Hispanic= 2 (1.4%), Middle Eastern= 1 (0.7%), Unknown= 11 (7.7%), No Intervention; African American= 59 (41.3%), Caucasian= 10 (7.0%), Hispanic= 2 (1.4%), Middle Eastern= 1 (0.7%), Unknown= 11 (7.7%), p = 0.776).

Active anticoagulation resulted in 45 (31.5%) patients requiring intervention vs 15 (10.5%) patients without anticoagulation requiring intervention, although this did not reach statistical significance (OR 1.08, 95% CI: 0.51 - 2.32; p = 0.85). A total of 71 (49.7%) of patients had nasal or facial oxygen use at the time of the epistaxis event. Notably, oxygen use showed a significantly decreased risk for intervention with 23 (16.1%) patients using oxygen requiring intervention and 37 (25.9%) patients not on oxygen requiring intervention (OR 0.45, 95% CI: 0.23 - 0.894; p = 0.028). As shown in Table 3, no other risk factors showed a significant difference between sample subgroups.

**Table 3.** Risk factors for epistaxis requiring intervention

**Risk Factor**	**Requiring intervention N (%)**	**OR (95% CI)**	**P-value**
**AC/AP Use**	45 (31.5%)	1.08 (0.51-2.32)	0.850
**Hypertension Diagnosis**	42 (29.4%	0.99 (0.50-1.96)	0.443
**Tobacco**	30 (21.0%)	1.13 (0.58-2.19)	0.722
**Intranasal medication**	30 (21.0%)	1.03 (0.53-1.99)	0.943
**Oxygen**	23 (16.1%)	0.45 (0.23-0.89)	**0.028**
**Alcohol**	26 (18.2%)	0.95(0.49-1.86)	0.882
**Renal Disease**	23 (16.1%)	0.99 (0.50-1.96)	0.979
**Previous Epistaxis **	18 (12.6%)	1.27 (0.60-2.66)	0.534
**Liver Disease **	10 (7.0%)	1.64 (0.62-4.34)	0.311
**Coagulopathy**	24 (16.8%)	0.87 (0.44-1.71)	0.733

AC/AP Use (Anticoagulation/Antiplatelet)

A final subgroup analysis demonstrated that 102 (71.3%) of sample patients with epistaxis required an alteration in medication management including holding anticoagulation, starting nasal saline spray, or nasal emollients.

## DISCUSSION

Our study sample included 143 hospitalized adults admitted for other clinical diseases that developed an epistaxis episode. Most sample patients, 104 (72.7%), had not been previously diagnosed with an episode of epistaxis. A significant portion of the patients, 106 (74.1%), had been using an anticoagulant medication and were diagnosed with hypertension 95 (66.4%).

Hypertension has long been a known risk factor for epistaxis.^5,10,12–15^ A meta-analysis performed by Jin Min et. al. 2017 on 10 studies showed an increased odds ratio for epistaxis in patients with hypertension (OR = 1.253; 95% CI: 1.080 - 1.453).^12^ A 2020 retrospective cohort study by Byun et. investigated the risk of hypertension in patients using a nationwide population cohort, demonstrating that hypertension was a significant risk factor for epistaxis with an adjusted hazard ratio of 1.47 (95% CI: 1.30 - 1.66) with hypertensive patients more likely to require posterior nasal packing.^13^

Another retrospective review by Sethi et. al. 2017 showed that patients with hypertension who presented to the emergency department were more likely to require nasal packing (41.2% vs 30.3%, p < 0.001).^16^ Our study showed that 95 (66.4%) patients who developed an episode of epistaxis did have hypertension. However, only 42 (29.4%) of our sample patients with hypertension required an intervention which was not significantly increased from the non-hypertensive patients.

Another notable risk factor identified in our sample was anticoagulant/antiplatelet medication use. We found that 106 (74.1%) post-admission epistaxis patients had been on anticoagulant/antiplatelet therapy. In addition, we also showed an overall (although not statistically significant) higher percentage of patients on anticoagulation/antiplatelet medications that required an epistaxis intervention (p = 0.850).

Previous studies have looked at the association of using different types of anticoagulant/antiplatelet medications and epistaxis risks, with classic medications (e.g., Warfarin Enoxaparin) significantly associated with more severe nasal bleeding than newer generation oral anticoagulants (e.g., Apixaban Xarelto).^17–19^

Our study results showed that 71 (49.7%) patients were on supplemental oxygen at time of their epistaxis episode. However, patients on oxygen were at a decreased risk for intervention compared to those not on oxygen. Although oxygen typically has been associated with nasal dryness when it is not humidified,^11^ a 2017 systematic review and meta-analysis demonstrated no statistically significant difference in the incidence of epistaxis among patients using non-humidified versus humidified low flow oxygen therapy.^19^

Finally, our study showed that most sample patients, 102 (71.3%), had an alteration in their medical management after their epistaxis episodes including discontinuing anticoagulants or addition of nasal saline sprays/nasal emollients. Although anticoagulants do increase the severity and frequency of epistaxis, other preventative and treatment measures should first be considered prior to discontinuation of these medications unless the bleeding is severe.^2^ However, our study results showed that some of the first treatments employed by setting providers to prevent epistaxis were discontinuing anticoagulant or antiplatelet therapy. Typically, nasal saline sprays and nasal emollients are recommended as first line preventative measures despite the lack of evidence showing any significant improvement with these moisturizing medications.^20,21^

### Study Limitations

One major limitation for our study was our retrospective design. Data obtained from this study was dependent on previously recorded EHR data. Data may have been omitted or inaccurately recorded which could affect the results of this study. For example, we acknowledge that providers did not always document the presence or absence of humidification for supplemental oxygen. This made it difficult for us to identify what forms of supplemental oxygen sample patients received that may have put them at varied risk for intervention.

## CONCLUSIONS

Based on these results, more systematically identifying at-risk patients who are admitted to the hospital can help to initiate preventative epistaxis measures. Our study demonstrated that the most common risk factors identified were hypertension and anticoagulation use. Most sample patients with epistaxis received an alteration of medical therapy including discontinuing anticoagulation and/or adding nasal sprays/nasal emollients. Further studies are needed to more comprehensively examine the numerous risk factors for epistaxis in the hospitalized patient populations and effective preventative measures for epistaxis.[Bibr ref-139976]

### Conflict of Interest

None
